# Differentiating high-grade patterns and predominant subtypes for IASLC grading in invasive pulmonary adenocarcinoma using radiomics and clinical-semantic features

**DOI:** 10.1186/s40644-025-00864-2

**Published:** 2025-03-28

**Authors:** Sunyi Zheng, Jiaxin Liu, Jiping Xie, Wenjia Zhang, Keyi Bian, Jing Liang, Jingxiong Li, Jing Wang, Zhaoxiang Ye, Dongsheng Yue, Xiaonan Cui

**Affiliations:** 1 Tianjin’s Clinical Research Center for Cancer, State Key Laboratory of Druggability Evaluation and Systematic Translational Medicine, Tianjin Key Laboratory of Digestive Cancer, Key Laboratory of Cancer Prevention and Therapy, Department of Radiology, Tianjin Medical University Cancer Institute and Hospital, National Clinical Research Center for Cancer, Tianjin, China; 2 Key Laboratory of Cancer Prevention and Therapy, Department of Lung Cancer, Tianjin Medical University Cancer Institute and Hospital, National Clinical Research Center of Cancer, Lung Cancer Center, Tianjin, China; 3 Department of Radiology, The Second Hospital of Shanxi Medical University, Taiyuan, China; 4 College of Computer Science and Technology, Zhejiang University, Hangzhou, China; 5 School of Public Health, Tianjin University of Traditional Chinese Medicine, Tianjin, China

**Keywords:** Adenocarcinoma of lung, Radiomics, Pathology, IASLC grading, Computed tomography

## Abstract

**Objectives:**

The International Association for the Study of Lung Cancer (IASLC) grading system for invasive non-mucinous adenocarcinoma (ADC) incorporates high-grade patterns (HGP) and predominant subtypes (PS). Following the system, this study aimed to explore the feasibility of predicting HGP and PS for IASLC grading.

**Materials and methods:**

A total of 529 ADCs from patients who underwent radical surgical resection were randomly divided into training and validation datasets in a 7:3 ratio. A two-step model consisting of two submodels was developed for IASLC grading. One submodel assessed whether the HGP exceeded 20% for ADCs, whereas the other distinguished between lepidic and acinar/papillary PS. The predictions from both submodels determined the final IASLC grades. Two variants of this model using either radiomic or clinical-semantic features were created. Additionally, one-step models that directly assessed IASLC grades using clinical-semantic or radiomic features were developed for comparison. The area under the curve (AUC) was used for model evaluation.

**Results:**

The two-step radiomic model achieved the highest AUC values of 0.95, 0.85, 0.96 for grades 1, 2, 3 among models. The two-step models outperformed the one-step models in predicting grades 2 and 3, with AUCs of 0.89 and 0.96 vs. 0.53 and 0.81 for radiomics, and 0.68 and 0.77 vs. 0.44 and 0.63 for clinical-semantics (*p* < 0.001). Radiomics models showed better AUCs than clinical-semantic models for grade 3 regardless of model steps.

**Conclusions:**

Predicting HGP and PS using radiomics can achieve accurate IASLC grading in ADCs. Such a two-step radiomics model may provide precise preoperative diagnosis, thereby supporting treatment planning.

**Supplementary Information:**

The online version contains supplementary material available at 10.1186/s40644-025-00864-2.

## Introduction

Lung cancer continues to be the foremost cause of cancer-related deaths globally [1]. Among its various forms, lung adenocarcinoma is the most prevalent histological subtype and has become the primary driver of lung cancer mortality. Although curative intent surgery can substantially improve survival rates or disease-specific outcomes [2–4], more than 30% of lung cancer patients still suffer from recurrence or die from their disease after surgery [5, 6].

The histological grading system is crucial for assessing the prognosis of patients with lung adenocarcinoma. The 2015 World Health Organization classification proposed five main histological subtypes that were closely associated with prognosis for grading lung adenocarcinoma patients [7–10]. However, this grading system may underestimate the impact of minor percentages of micropapillary, secondary subtypes and complex glandular patterns which can also significantly influence patient outcomes [10–12]. Considering this limitation, the International Association for the Study of Lung Cancer (IASLC) introduced a novel grading system that considered both predominant subtypes and the proportion of high-grade patterns regarding micropapillary, solid, and complex glandular [13]. Recent studies had evaluated this new grading system [14, 15]. Deng et al. found that patients with stage IB to III high-grade invasive non-mucinous adenocarcinomas exhibited an improved survival rate when treated with adjuvant chemotherapy [14]. The study by Rokutan-Kurata et al. showed a concordance index of 0.77 for assessing recurrence risk in stage I-IIIA lung adenocarcinoma patients using the IASLC grading system [15]. These results indicated that the IASLC system, which used primary subtypes with a threshold of 20% to define high-grade patterns, was superior for guiding postoperative prognosis.

In addition to the benefits of analysing postoperative pathology for prognosis, understanding histological subtypes before surgery is also of importance to guide preoperative treatment planning [16]. However, preoperative biopsy is limited by tumour heterogeneity and sampling constraints, making it challenging to comprehensively assess pathological subtypes. As the most commonly used method for preoperative evaluation of lung nodules, CT imaging can provide detailed morphological information. Building on this, the development of an accurate preoperative imaging-based model using CT scans to predict IASLC grades might serve as a valuable reference for clinical decisions regarding neoadjuvant therapy and lymph node dissection before surgery, potentially improving patient outcomes. For example, in patients with stage IB lung adenocarcinoma, those classified as IASLC grade 3 postoperatively are often advised to undergo chemotherapy or radiotherapy to reduce the risk of recurrence. By identifying patients who are likely to have IASLC grade 3 before surgery, the model can support clinical decision-making regarding neoadjuvant therapy.

To this end, recent studies have focused on using CT-based models for non-invasive IASLC grading [17, 18]. For instance, Yang et al. combined radiomics and semantic features on CT scans to create a nomogram that distinguished IASLC grades 1 and 2 from high-grade patients, achieving an AUC of 0.84 on the validation set [17]. Another study employed radiomic features to develop a model that attained an AUC of 0.90 in differentiating grade 1 from grades 2 and 3 [18]. These models classified patients into IASLC grades directly without predicting predominant subtypes and the proportion of high-grade patterns. While they showed acceptable performance in IASLC grading, incorporating the classification of predominant subtypes and estimating the proportion of high-grade patterns according to the IASLC system might further enhance grading accuracy.

Therefore, our research aimed to explore the effectiveness of predicting high-grade patterns and predominant subtypes in two steps for IASLC grading in invasive non-mucinous pulmonary adenocarcinoma using radiomic or clinical-semantic features. The study was also designed to compare these two-step models with one-step models that directly assessed IASLC grades using clinical-semantic or radiomic features. The ultimate goal was to accurately identify high-grade patients, thereby supporting preoperative treatment planning.

## Materials and methods

### Study participants

This retrospective study involved 958 consecutive patients who underwent radical surgical resection and presented with lung adenocarcinoma from Tianjin Medical University Cancer Institute and Hospital between April 2022 and December 2023. The study received approval from the medical ethical committee of the hospital (EK2023044). The inclusion criteria were: (1) thin-slice, plain chest CT images taken within one month before surgery; (2) detailed postoperative pathology reports with information on the proportion of pathological subtypes; (3) having received no treatment before surgery. The exclusion criteria were: (1) poor quality CT images; (2) without preoperative CT images within one month; (3) without clear histopathological subtypes; (4) precursor glandular lesions, minimally invasive adenocarcinoma or invasive mucinous adenocarcinoma [13].

### Histopathology assessment

Two pathologists were involved in assessing and classifying invasive non-mucinous pulmonary adenocarcinoma, one with 3 years of experience and the other with 10 years of experience. The initial pathological analysis was performed by the junior pathologist, followed by a review and final confirmation by the senior pathologist to ensure diagnostic accuracy. The pathological classification followed the 5th edition of the World Health Organization classification of thoracic tumours [19]. Specifically, the diagnosis of invasive non-mucinous pulmonary adenocarcinoma involved a detailed examination of histologic patterns, categorized into lepidic, acinar, papillary and high-grade patterns including micropapillary, solid, and complex glandular [20]. They documented the proportion of each pathological subtype in increments of 5% and assigned grades based on the IASLC grading system [15]. Grade 1 represented a lepidic predominant pattern with no or less than 20% of high-grade components; Grade 2 denoted an acinar or papillary predominant pattern with no or less than 20% of high-grade components; Grade 3 corresponded to any tumour with 20% or more of high-grade components.

### CT examination and clinical-semantic features

Low-dose chest CT scans were performed using GE Discovery CT750 HD, GE LightSpeed 16, and Siemens Somatom Sensation 64 CT devices. The scanning range was from the lung apex to below the diaphragm. The tube voltage was 120 kV, and the tube current was automatically adjusted. The reconstruction thickness was 1.25 mm with a spacing of 0.984 mm in the GE CT system, whereas the reconstruction thickness was 1.5 mm with a spacing of 0.95 mm for the Siemens CT device. Lung nodules on CT scans for each patient were independently evaluated by two experienced radiologists. During the evaluation, the radiologists were blinded to the histopathological results, and any discrepancies were resolved through mutual consultation. After evaluation, fourteen clinical-semantic features were recorded. The specific characteristics were as follows: (1) age, (2) gender, (3) nodule diameters averaged from the long-axis and short-axis diameters, (4) nodule types, (5) nodule location, (6) shape, (7) lobulation, (8) spiculation, (9) cavitation, (10) vacuole, (11) air bronchograms, (12) pleural traction, (13) vascular convergence, and (14) obstructive pneumonia.

### Nodule delineation and radiomics feature extraction

**Fig. 1:**
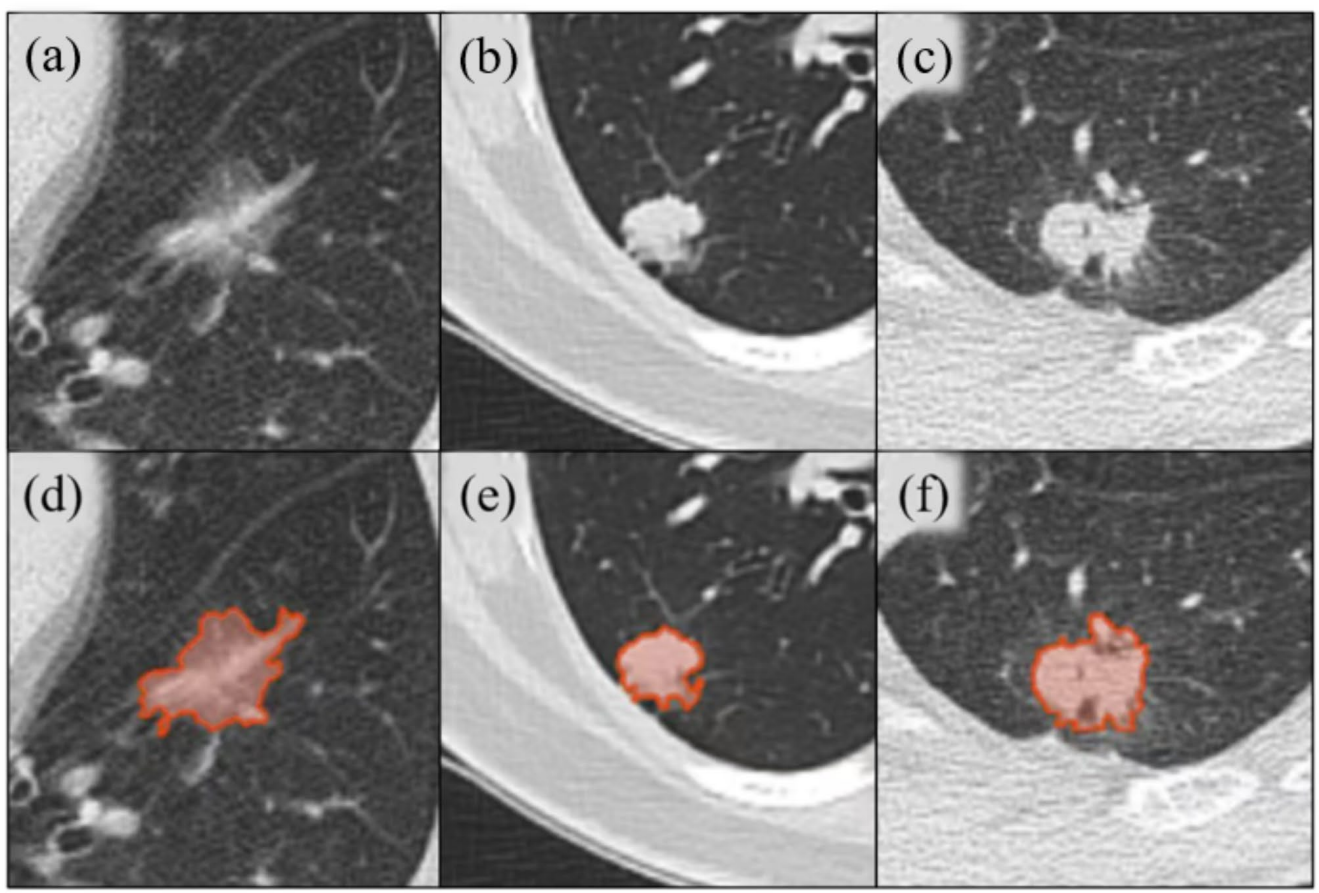
Examples of invasive non-mucinous pulmonary adenocarcinoma with delineations. (**a**) A 72-year-old patient classified as IASLC 1; (**b**) A 43-year-old female patient classified as IASLC 2; (**c**) A 56-year-old male patient classified as IASLC 3; (d)-(f) Delineation examples corresponding to (**a**)-(**c**)

The region of interest for each pulmonary lesion was semi-automatically delineated using the Samm Base plugin (https://github.com/bingogome/samm) in 3D Slicer software (Version 5.6.2). The examples of delineations are shown in Fig. 1. Two radiologists evaluated the segmentation results and manually adjusted the delineations. Before radiomics feature extraction, delineated CT data was resampled to 1 × 1 × 1 mm voxel size to ensure consistent spatial resolution and improve the robustness of the extracted features. Pyradiomics (Version 3.1.0) was then applied to extract radiomics features including first-order features, shape features, grey level co-occurrence matrix features, grey level size zone matrix features, grey level run length matrix features, grey level dependence matrix features, and neighbourhood grey tone difference matrix features. As a consequence, a total of 1834 radiomics features were obtained after extraction. To find robust radiomics features for model development, we conducted a consistency evaluation between radiologists using the intraclass correlation coefficient (ICC). A subset comprising one-third of the cases was randomly selected. After two month, intra-observer and inter-observer segmentation reproducibility were performed on the subset by two radiologists separately [17]. Ultimately, 1465 radiomics features with an ICC > 0.75 were retained for the experiment [21].

### Model development

**Fig. 2:**
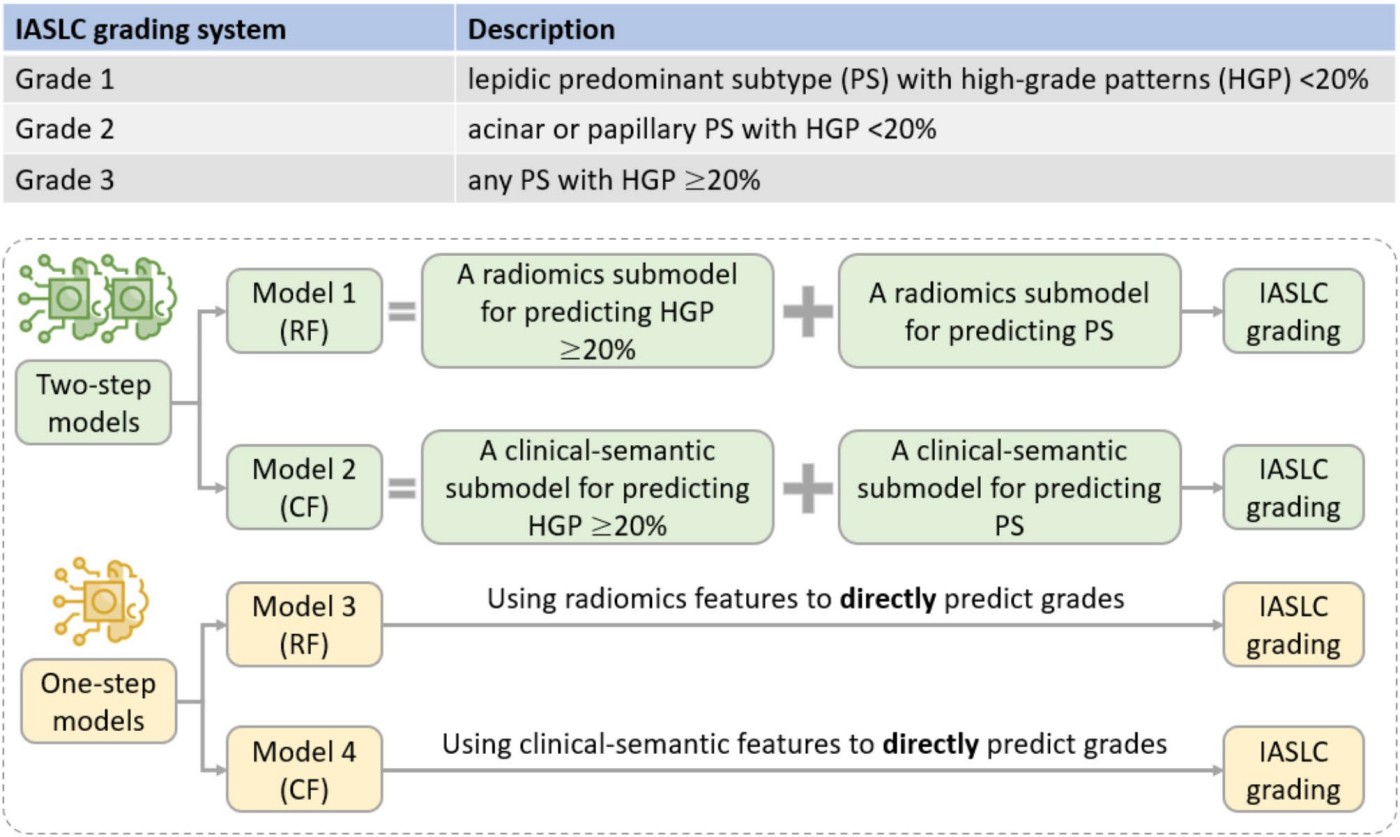
Concepts of two-step and one-step models. Two-step models using radiomics features (RF) or clinical-semantic features (CF) to classify IASLC grades through the prediction of high-grade patterns and predominant subtypes, whereas one-step models directly assessed IASLC grades using RF or CF

In order to explore the feasibility of predicting high-grade patterns and predominant subtypes for IASLC grading, we developed two distinct two-step models, with one based on radiomic features and the other on clinical-semantic features. To further evaluate model effectiveness, we compared these two-step models with two one-step models that directly assessed IASLC grades using either clinical-semantic or radiomic features. The detailed development process of the two-step and one-step models is outlined below. The basic concepts of the four models are shown in Fig. 2.

#### Development of two-step models

the two-step model for IASLC grading was developed using two submodels. The first submodel assessed whether the proportion of high-grade patterns was ≥ 20%, while the second submodel differentiated between lepidic and acinar/papillary predominant subtypes. The IASLC grading of invasive pulmonary adenocarcinoma was then determined based on the estimations from these two submodels. In this analysis, two types of two-step models were established. One model consisted of two submodels that used clinical-semantic features as input, whereas the other type also comprising two submodels relied on radiomic features derived from nodule delineation.

The submodels using clinical-semantic were built using weighted logistic regression, where the class weight was calculated as the total sample size divided by the product of the sample size of a specific class and the total number of classes. Classes with larger quantities were assigned smaller weights, while those with smaller quantities were assigned larger weights. The introduced class weights can mitigate the impact of the data imbalance on model performance. Variates including age, gender, diameter and semantic CT features were used. Univariate analysis was applied to preliminarily identify potential predictive features. Any input feature with a p-value smaller than 0.1 in the univariate analysis was included for further analysis [22]. Subsequently, we conducted a multivariate analysis using the backward likelihood ratio method to construct the final submodels.

Regarding the submodels using radiomics features, minimum redundancy maximum relevance (mRMR), least absolute shrinkage and selection operator (LASSO), and logistic regression methods were applied. Initially, all radiomics features were standardized using the Z-score normalization method. The mRMR approach was then employed to process the radiomics features, identifying the top 30 most significant features with the highest correlation to the outcomes. Subsequently, the LASSO algorithm, combined with ten-fold cross-validation, was used to determine the optimal subset of predictive features and their corresponding coefficients. These optimal features were used to construct the radiomics submodels via weighted binary logistic regression. For each lung nodule, the radiomics feature score (Rad-score) of a submodel was derived by linearly combining the selected features, weighted by their respective coefficients.

#### Development of one-step models

for comparisons, we also developed two types of one-step models that used only clinical-semantic or radiomics features for IASLC grading. The model using clinical-semantic features was built using ordinal logistic regression with class weights to alleviate data imbalance. Univariate ordinal logistic regression with a proportional odds ratio was first applied to select potential features for predicting IASLC grades. Then the multivariate ordinal logistic regression with stepwise selection was employed to construct the final one-step clinical-semantic model. In contrast, the one-step model using radiomics features was developed by using the mRMR, LASSO, and multivariate ordinal logistic regression methods with class weights to mitigate data imbalance. Compared to the two-step models, the one-step models directly assessed IASLC grades without predicting the proportion of high-grade patterns or predominant subtypes.

### Statistical analysis and evaluation metrics

Statistical analyses were conducted using Python (version 3.11.7). Group comparisons for categorical variables in the training and validation sets were performed using the chi-squared test, while continuous variables were analyzed using either the t-test or the Kruskal-Wallis H test [23–25], depending on the data distribution. Model performance was evaluated by comparing the area under the curve (AUC), sensitivity, specificity and F1 scores. The optimal threshold in the receiver operating characteristic curve for classification was determined by selecting the threshold that maximized the sum of sensitivity and specificity. At this optimal threshold, sensitivity was calculated as the ratio of true positives to the sum of true positives and false negatives, while specificity was calculated as the ratio of true negatives to the sum of true negatives and false positives. The DeLong test applied to assess differences in AUC between models [26]. The Cohen’s kappa test was used to assess the classification agreement between the models and the pathologist’s IASLC grading. The clinical utility of the model was evaluated through decision curve analysis, which involved assessing net benefits across a range of threshold probabilities in the validation cohort. A two-tailed p-value of less than 0.05 was considered statistically significant.

## Results

### Patient characteristics

**Fig. 3:**
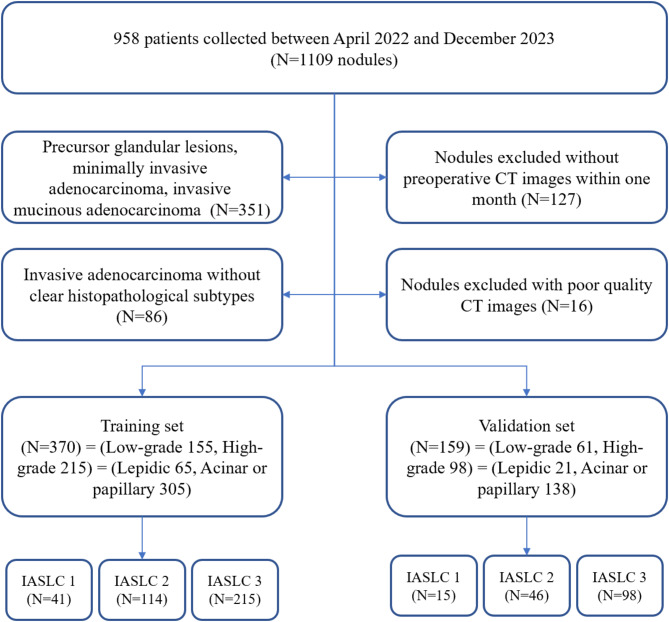
Flow chart of data selection in the study

After data selection, 529 lung nodules remained in the study. The lung nodules were randomly divided into training and validation sets in a 7:3 ratio. The detailed flow chart for data selection is shown in Fig. 3. Patient characteristics in the training and validation sets for IASLC grading are shown in Table 1. Patient characteristics for the prediction of high-grade patterns and predominant subtypes are described in Tables S1 and S2. The inter-reader agreement for semantic feature classifications are shown in Table S3. For IASLC grading, significant differences were observed in diameter, types, spiculation, air bronchograms, pleural traction and obstructive pneumonia among the three groups in the training and validation datasets. There was no significant difference found in cavitation and vascular convergence among the groups in both datasets.

**Table 1:** Patient characteristics in the training and validation sets for IASLC grading

Variables			Training set (*n* = 370)			Validation set (*n* = 159)		
	IASLC 1	IASLC 2	IASLC 3	*P* value	IASLC 1	IASLC 2	IASLC 3	*P* value
Age (years)	56.512 ± 9.02	58.877 ± 8.878	60.521 ± 9.806	0.017	56.933 ± 10.826	57.543 ± 8.831	58.939 ± 8.119	0.716
Gender				0.046				0.288
Male	22	51	127		6	13	41	
Female	19	63	88		9	33	57	
Diameter (cm)	1.82 ± 0.697	1.864 ± 0.973	2.445 ± 1.102	$\:<$0.001	1.377 ± 0.365	1.792 ± 0.922	2.368 ± 0.991	$\:<$0.001
Types				$\:<$0.001				$\:<$0.001
Solid	3	85	187		1	30	80	
Subsolid	38	29	28		14	16	18	
Nodule location				0.035				0.051
Peripheral	41	110	196		15	46	89	
Central	0	4	19		0	0	9	
Shape				0.179				0.002
Irregular	18	35	63		11	16	27	
Regular	23	79	152		4	30	71	
Lobulation				0.343				0.001
No	16	40	63		12	15	30	
Yes	25	74	152		3	31	68	
Spiculation				0.001				$\:<$0.001
No	26	59	77		13	24	29	
Yes	15	55	138		2	22	69	
Cavitation				0.088				0.231
No	41	108	196		15	40	91	
Yes	0	6	19		0	6	7	
Vacuole				0.017				0.162
No	33	101	162		11	42	79	
Yes	8	13	53		4	4	19	
Air bronchograms				0.003				0.048
No	39	79	152		14	40	71	
Yes	2	35	63		1	6	27	
Pleural traction				0.004				0.010
No	18	49	57		9	17	23	
Yes	23	65	158		6	29	75	
Vascular convergence				0.357				0.926
No	14	53	98		7	20	46	
Yes	27	61	117		8	26	52	
Obstructive pneumonia				0.001				0.018
No	41	109	184		15	46	86	
Yes	0	5	31		0	0	12	

### Two-step models

In the development of clinical-semantic submodels in two-step models, univariable analysis showed that age, gender, diameter, types, nodule location, spiculation, cavitation, vacuole, pleural traction, obstructive pneumonia had the potential to be predictive in assessing whether the proportion of high-grade patterns was greater than or equal to 20% in invasive non-mucinous pulmonary adenocarcinoma. In contrast, age, diameter, types, and air bronchograms had potential be predictive for the discrimination between lepidic and acinar/papillary predominant subtypes. After performing multivariate logistic regression analysis, diameter, types, spiculation and vacuole emerged as statistically significant features for high-grade pattern prediction, whereas types was the only significant feature for predominant subtype prediction. The detailed feature selection results univariate and multivariate analysis are shown in Tables S4 and S5.

In the development of radiomics submodels in two-step models, the analysis of the mRMR, LASSO and multivariate binary logistic regression approaches found four features (wavelet-LLL_gldm_LargeDependenceHighGrayLevelEmphasis, lbp-2D_firstorder_Entropy, logarithm_ngtdm_Strength, exponential_firstorder_RobustMeanAbsoluteDeviation) were identified as statistically significant for the prediction of high-grade patterns greater than or equal to 20%. In contrast, a similar set of six features (exponential_firstorder_RobustMeanAbsoluteDeviation, wavelet-LLL_gldm_LargeDependenceHighGrayLevelEmphasis. logarithm_firstorder_Skewness, logarithm_glcm_DifferenceAverage, square_gldm_LargeDependenceLowGrayLevelEmphasis, logarithm_glcm_Idn) were selected to construct the radiomics submodel for the prediction of predominant subtypes. These radiomics features were used to build calculation equations for the prediction scores of high-grade patterns and predominant subtypes, which were presented in the supplementary file. Besides, we also tried to combine both significant clinical-semantic features and radiomic prediction scores to build submodels for both tasks employing using logistic regression with backward selection. However, only radiomics prediction scores were retained.

### One-step models

For the one-step model using clinical-semantic features, the univariable ordinal logistic regression analysis showed that age, gender, diameter, types, nodule location, spiculation, cavitation, vacuole, air bronchograms, pleural traction, obstructive pneumonia were independent variables for differentiating IASLC grading. The final model only included types after the stepwise multivariable ordinal logistic regression analysis. Regarding the one-step model using radiomics features, the analysis of the mRMR, LASSO and multivariate ordinal logistic regression methods selected the predictive radiomics features including exponential_firstorder_RobustMeanAbsoluteDeviation, wavelet-LLL_glrlm_HighGrayLevelRunEmphasis, and logarithm_firstorder_Skewness to construct the final model for IASLC grading. The parallel regression assumption was also not violated. The Rad-score in the one-step model using radiomics features was shown in the supplementary.

### Performance of submodels in two-step models

**Fig. 4:**
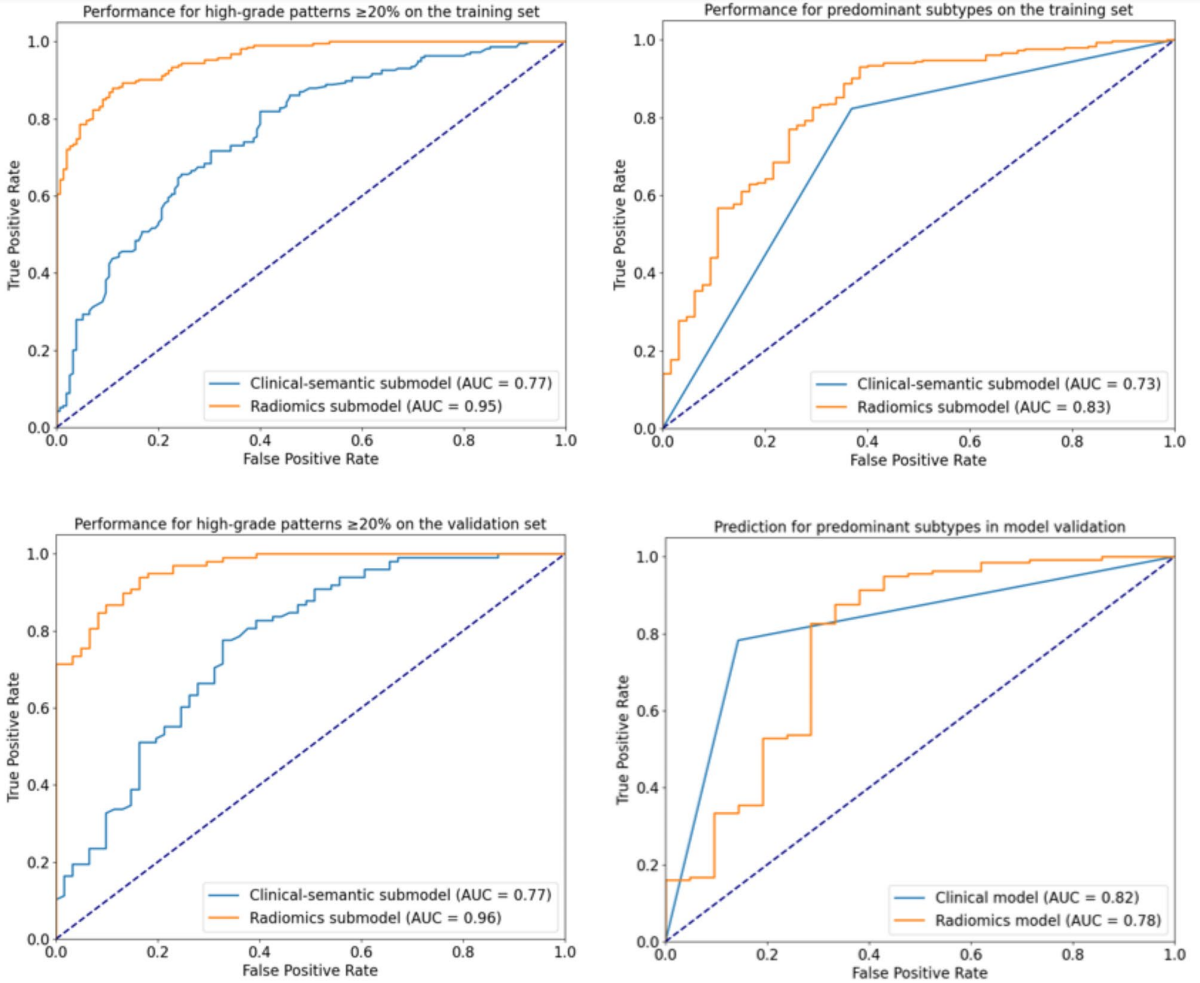
Receiver operating characteristic curves of the submodels for predicting high-grade patterns $\:\ge\:$20% and predominant subtypes in invasive non-mucinous pulmonary adenocarcinoma

The AUC performance of the clinical-semantic and radiomics submodels in the two-step models are shown in Fig. 4. The radiomics submodel had significantly higher AUCs than the clinical-semantic submodel for high grade pattern prediction (0.95 vs. 0.77, *p* < 0.001; 0.96 vs. 0.77, *p* < 0.001) in both the training and validation sets. For the prediction of predominant subtypes, the radiomics submodel significantly outperformed the clinical-semantic submodel in the training set and showed no significant difference in performance on the validation set (*p* = 0.52).

### Performance comparison between two-step and one-step models

We calculated the performance of the two-step and one-step models using either radiomics features or clinical-semantic features for IASLC grading, as shown in Table 2. When differentiating IASLC grades, the two-step model using radiomics features yielded the highest AUC values. The AUCs for the models using radiomics features in grade 3 were higher than those for the models using clinical-semantic features regardless of the model steps. In the comparison of one-step and two-step models using radiomics features for IASLC grading, the two-step model was found to outperform the one-step model in predicting grades 2 and 3 (0.88 vs. 0.63, *p* < 0.001, 0.95 vs. 0.82, *p* < 0.001; 0.85 vs. 0.53, *p* < 0.001; 0.96 vs. 0.81, *p* < 0.001) in both datasets. Similarly, the two-step model had significantly better performance than the one-step model when using clinical-semantic features as input for the classification of grades 2 and 3 (all *p* < 0.001). We also assessed the classification agreement between the models and the pathologist’s IASLC grading in Table 2. The table showed that compared to the two-step model using clinical-semantic features or the one-step models, the two-step model using radiomics achieved an overall higher classification agreement with the pathologist in IASLC grading. The agreement value of the two-step radiomics model exceeded 0.7 for grade 3, indicating the potential of the model for accurately identifying high-grade cases and assisting in preoperative treatment planning.

**Table 2:** Diagnostic performance of one-step and two-step models for IASLC grading. One-step and two-step models used either radiomics features (RF) or clinical-semantic features (CF) as input. The highest AUC values along with 95% their confidence intervals (CI) are shown in bold. The Cohen’s kappa test was used to assess the classification agreement between the models and the pathologist’s IASLC grading

Model variants	IASLC grades	Training set					Validation set				
		AUC(95%CI)	Sensitivity	Specificity	F1	Kappa	AUC(95%CI)	Sensitivity	Specificity	F1	Kappa
Two-step (RF)	1	**0.96(0.94–0.98)**	0.98	0.87	0.65	0.58	**0.95(0.91–0.99)**	0.87	0.85	0.53	0.46
	2	**0.88(0.84–0.91)**	0.47	0.92	0.57	0.44	**0.85(0.80–0.91)**	0.37	0.89	0.45	0.30
	3	**0.95(0.93–0.97)**	0.88	0.85	0.89	0.73	**0.96(0.94–0.98)**	0.89	0.85	0.90	0.74
One-step (RF)	1	0.95(0.91–0.97)	0.93	0.90	0.68	0.63	0.92(0.85–0.98)	0.80	0.88	0.55	0.48
	2	0.63(0.57–0.69)	0.36	0.83	0.41	0.20	0.53(0.42–0.64)	0.37	0.79	0.39	0.16
	3	0.82(0.77–0.86)	0.78	0.70	0.78	0.48	0.81(0.73–0.88)	0.74	0.74	0.78	0.47
Two-step (CF)	1	0.91(0.87–0.95)	0.93	0.84	0.58	0.50	0.92(0.87–0.97)	0.74	1.00	0.44	0.34
	2	0.66(0.60–0.72)	0.66	0.59	0.51	0.21	0.68(0.58–0.77)	0.86	0.50	0.54	0.38
	3	0.77(0.71–0.81)	0.82	0.60	0.78	0.43	0.77(0.68–0.84)	0.67	0.78	0.78	0.44
One-step (CF)	1	0.90(0.85–0.94)	0.90	0.88	0.64	0.58	0.88(0.80–0.93)	0.93	0.76	0.44	0.35
	2	0.53(0.47–0.58)	0.31	0.75	0.33	0.05	0.44(0.35–0.53)	0.07	0.94	0.11	0.00
	3	0.67(0.62–0.73)	0.93	0.38	0.78	0.33	0.63(0.54–0.72)	0.82	0.49	0.77	0.32

**Fig. 5:**
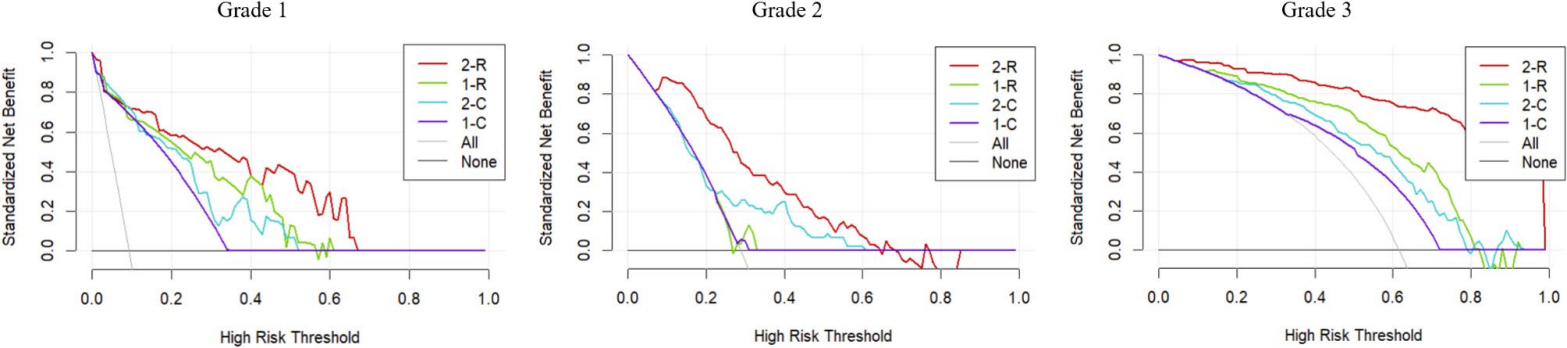
Decision curve analysis for different models. 2-R, 1-R, 2-C, 1-C represent two-step radiomics, one-step radiomics, two-step clinical-semantics and one-step clinical-semantics models

For the three IASLC grades, the two-step radiomics model showed better net benefit than the other models, as shown in Fig. 5. In particular, the two-step radiomics model for grade 3 achieved good net benefits across various thresholds, highlighting its potential to identify high-grade patients and providing valuable insights for clinical decisions regarding neoadjuvant therapy and lymph node dissection before surgery. Additionally, the two-step models, whether using radiomics or clinical-semantics features, outperformed the one-step models when using the same input features. This may reflect the superiority of predicting high-grade patterns and predominant subtypes in two steps for IASLC grading.

## Discussion

We developed a two-step radiomics model for the differentiation of IASLC grades in invasive non-mucinous pulmonary adenocarcinoma. The model yielded accurate results on the prediction of high grade patterns > 20% and predominant subtypes, which results in high AUC scores of 0.95, 0.85 and 0.96 on the validation set for three category IASLC grading. The results suggested that the proposed model may assist radiologists in the stratification of patients according to different IASLC grades, thereby supporting treatment planning prior to surgery.

Studies have shown that radiomics features on CT images can describe predict the presence of high-grade patterns [8, 27]. For example, Chen et al. utilized cases with nearly pure lung adenocarcinoma to develop a radiomics method [27]. The method can assess micropapillary and solid components using patch-wise image analysis with an AUC of 0.86. Another study built and compared four machine learning algorithms with radiomics features for the prediction of presence of micropapillary or solid patterns [28]. The best performed algorithm achieved an AUC of 0.75 in the internal validation. Compared to their studies, our radiomics submodel yielded a higher AUC of 0.96 on the validation set. This might be explained by the fact that predicting high-grade patterns > 20% was easier than assessing the presence of high-grade patterns. Other researchers also studied if the radiomics method was able to predict predominant subtypes. The research from Yang et al. showed radiomics features can be used to stratify near-pure lepidic, papillary, acinar, micropapillary, and solid subtypes in lung adenocarcinoma [29]. Similarly, Park et al. found that using CT-based radiomics features differentiated the predominant lepidic subtype from acinar or papillary subtypes [30], achieving an AUC of 0.86, which was comparable to the AUC of our radiomics submodel at 0.78. Unlike their studies, we further utilized the prediction results of predominant subtypes along with the information regarding whether high-grade patterns exceeded 20% to achieve IASLC grading.

Accurate IASLC grading of lung adenocarcinoma preoperative is essential to patients since it not only helps predict patient prognosis but also enables the formulation of the treatment approach. To attain this goal, Li proposed an ordinal radiomics method utilizing 682 nodules for IASLC grading. Their method was considered as a one-step model and tended to misclassify the grade 2 to grades 1 or 3 [18]. This is consistent with the results of the one-step radiomics model as illustrated in the model performance comparison. However, though a two-step strategy, our model using radiomics features was able to not maintain the high AUCs of grades 1 and 3, but also improve the performance in grade 2. In addition, our two-step radiomics model outperformed another radiomics method that was designed to directly select grade 3 patients [17], with AUC values of 0.95 vs. 0.82 and 0.96 vs. 0.81 on the training and validation sets, respectively. These findings somehow showed the superiority of our proposed two-step model with radiomics features for IASLC grading. In addition to our two-step radiomics model, deep learning can also have good performance in the classification of IASLC grades. For example, a study employed deep learning models including ResNet, VGG, and InceptionNet to perform IASLC grading [31]. Trained on CT data from 339 patients with invasive adenocarcinoma, their deep learning system achieved AUC values of 0.93, 0.80, and 0.95 for grades 1, 2, and 3 on a test set of 76 patients. However, deep learning often faces challenges in the interpretability of its predictions. In contrast, radiomics can provide a rad_score equation incorporating first-order features, which describe the statistical distribution of pixel intensities and transform-based features, such as wavelet-transformed characteristics, which capture multi-scale image information. By integrating these features, radiomics models can enhance interpretability, making them more accessible for both clinicians and patients and facilitating their clinical application.

We also explored the impact of CT acquisition variability on model performance. Table S7 shows that there was no difference in the AUC values of the two-step radiomics model between the two vendor settings. However, the F1 score of the model for grade 1 in the GE scans was significantly higher than that of the model in the Siemens scans. This might suggest that including more Siemens scans on grade 1 is needed to improve model performance.

The study also had some limitations. First, the data used in this study was obtained from a single centre. Difference in population, parameters of CT scanners and image reconstruction algorithms may affect the performance of the model applied on other cohorts. In the future study, we will collect data from other institutions to further optimize the model and validate it across diverse patient populations. Second, The study retrospectively collected data to develop a model for predicting IASLC grading. Further investigation is warranted to assess the accuracy of the model in prospective patient cohorts and to explore the correlation between grading predictions and patient outcomes. Third, we only considered semantic CT features and patient information including age and gender to develop the clinical-semantic models. Integrating other data, such as smoking history, lymphovascular invasion, and genomic alterations, may provide added value for better reflecting the relationship between invasive pulmonary adenocarcinoma and IASLC grades [32].

## Conclusion

In conclusion, through the prediction of predominant subtypes and high-grade patterns, we presented a two-step radiomics model for the differentiation of IASLC grading in invasive pulmonary adenocarcinoma. The model had potential to provide accurate IASLC grading for patients before surgery.

## Electronic supplementary material

Below is the link to the electronic supplementary material.

Supplementary Material 1

## Data Availability

No datasets were generated or analysed during the current study.

## Declarations

### Abbreviations

IASLC International Association for the Study of Lung Cancer Pathology Committee.

ADC invasive non-mucinous adenocarcinoma.

PS predominant subtypes.

HGP high-grade patterns.

AUC Area Under the Curve.

ICC Intraclass Correlation Coefficient.

mRMR Minimum Redundancy Maximum Relevance.

LASSO Least Absolute Shrinkage and Selection Operator.

OR Odds Ratio.

CI Confidence Interval.

### Author details

^1^Tianjin Medical University Cancer Institute and Hospital, National Clinical Research Center for Cancer, Tianjin’s Clinical Research Center for Cancer, State Key Laboratory of Druggability Evaluation and Systematic Translational Medicine, Tianjin Key Laboratory of Digestive Cancer, Key Laboratory of Cancer Prevention and Therapy, Department of Radiology, Tianjin, China. ^2^Tianjin Medical University Cancer Institute and Hospital, Key Laboratory of Cancer Prevention and Therapy, National Clinical Research Center of Cancer, Department of Lung Cancer, Lung Cancer Center, Tianjin, China. ^3^The Second Hospital of Shanxi Medical University, Department of Radiology, Taiyuan, China. ^4^Zhejiang University, College of Computer Science and Technology, Hangzhou, China. ^5^Tianjin University of Traditional Chinese Medicine, School of Public Health, Tianjin, China.
