# Supplementary material for: Differentiating high-grade patterns and predominant subtypes for IASLC grading in invasive pulmonary adenocarcinoma using radiomics and clinical-semantic features
Source: Cancer Imaging. 2025 Mar 28;25:42. doi: 10.1186/s40644-025-00864-2 (PMC11951669; doi:10.1186/s40644-025-00864-2)
Supplement: Supplementary file 1 — Supplementary Material 1 [file 40644_2025_864_MOESM1_ESM.docx]

**Appendix**

Table S1 Patient Characteristics in the training and validation sets for high-grade pattern prediction.

| Variables | Training set (n=370) | | | Validation set (n=159) | | |
| --- | --- | --- | --- | --- | --- | --- |
|  | $<$ 20% | $\geq$ 20% | P value | $<$ 20% | $\geq$ 20% | P value |
| Age (years) | 58.252±8.948 | 60.521±9.806 | 0.014 | 57.393±9.269 | 58.939±8.119 | 0.442 |
| Gender |  |  | 0.023 |  |  | 0.176 |
| Male | 73 | 127 |  | 19 | 41 |  |
| Female | 82 | 88 |  | 42 | 57 |  |
| Diameter (cm) | 1.852±0.906 | 2.445±1.102 | $<$0.001 | 1.69±0.837 | 2.368±0.991 | $<$0.001 |
| Types |  |  | $<$0.001 |  |  | $<$0.001 |
| Solid | 88 | 187 |  | 31 | 80 |  |
| Subsolid | 67 | 28 |  | 30 | 18 |  |
| Nodule location |  |  | 0.014 |  |  | 0.037 |
| Peripheral | 151 | 196 |  | 61 | 89 |  |
| Central | 64 | 19 |  | 0 | 9 |  |
| Shape |  |  | 0.317 |  |  | 0.030 |
| Irregular | 53 | 63 |  | 27 | 27 |  |
| Regular | 102 | 152 |  | 34 | 71 |  |
| Lobulation |  |  | 0.165 |  |  | 0.081 |
| No | 56 | 63 |  | 27 | 30 |  |
| Yes | 99 | 152 |  | 34 | 68 |  |
| Spiculation |  |  | $<$0.001 |  |  | $<$0.001 |
| No | 85 | 77 |  | 37 | 29 |  |
| Yes | 70 | 138 |  | 24 | 69 |  |
| Cavitation |  |  | 0.060 |  |  | 0.760 |
| No | 149 | 196 |  | 55 | 91 |  |
| Yes | 6 | 19 |  | 6 | 7 |  |
| Vacuole |  |  | 0.008 |  |  | 0.306 |
| No | 134 | 162 |  | 53 | 79 |  |
| Yes | 21 | 53 |  | 8 | 19 |  |
| Air bronchograms |  |  | 0.246 |  |  | 0.016 |
| No | 118 | 152 |  | 54 | 71 |  |
| Yes | 37 | 63 |  | 7 | 27 |  |
| Pleural traction |  |  | 0.001 |  |  | 0.011 |
| No | 67 | 57 |  | 26 | 23 |  |
| Yes | 88 | 158 |  | 35 | 75 |  |
| Vascular convergence |  |  | 0.653 |  |  | 0.742 |
| No | 67 | 98 |  | 27 | 46 |  |
| Yes | 88 | 117 |  | 34 | 52 |  |
| Obstructive pneumonia |  |  | $<$0.001 |  |  | 0.011 |
| No | 150 | 184 |  | 61 | 86 |  |
| Yes | 5 | 31 |  | 0 | 12 |  |

Table S2 Patient Characteristics in the training and validation sets for predominant subtype prediction.

| Variables | Training set (n=370) | | | Validation set (n=159) | | |
| --- | --- | --- | --- | --- | --- | --- |
|  | Lepidic | Acinar or papillary | P value | Lepidic | Acinar or papillary | P value |
| Age (years) | 57.6±8.923 | 59.99±9.591 | 0.041 | 58±10.025 | 58.399±8.382 | 0.929 |
| Gender |  |  | 0.609 |  |  | 0.603 |
| Male | 37 | 163 |  | 9 | 51 |  |
| Female | 28 | 142 |  | 12 | 87 |  |
| Diameter (cm) | 1.921±0.707 | 2.255±1.118 | 0.050 | 1.507±0.431 | 2.199±1.019 | 0.001 |
| Types |  |  | $<$0.001 |  |  | $<$0.001 |
| Solid | 24 | 251 |  | 3 | 108 |  |
| Subsolid | 41 | 54 |  | 18 | 30 |  |
| Nodule location |  |  | 0.151 |  |  | 0.485 |
| Peripheral | 64 | 283 |  | 21 | 129 |  |
| Central | 1 | 22 |  | 0 | 9 |  |
| Shape |  |  | 0.174 |  |  | $<$0.001 |
| Irregular | 25 | 91 |  | 15 | 39 |  |
| Regular | 40 | 214 |  | 6 | 99 |  |
| Lobulation |  |  | 0.540 |  |  | 0.002 |
| No | 23 | 96 |  | 14 | 43 |  |
| Yes | 42 | 209 |  | 7 | 95 |  |
| Spiculation |  |  | 0.211 |  |  | 0.012 |
| No | 33 | 129 |  | 14 | 52 |  |
| Yes | 32 | 176 |  | 7 | 86 |  |
| Cavitation |  |  | 0.303 |  |  | 0.853 |
| No | 63 | 282 |  | 20 | 126 |  |
| Yes | 2 | 23 |  | 1 | 12 |  |
| Vacuole |  |  | 0.733 |  |  | 0.560 |
| No | 51 | 245 |  | 16 | 116 |  |
| Yes | 14 | 60 |  | 5 | 22 |  |
| Air bronchograms |  |  | 0.043 |  |  | 0.571 |
| No | 54 | 216 |  | 10 | 39 |  |
| Yes | 11 | 89 |  | 11 | 99 |  |
| Pleural traction |  |  | 0.821 |  |  | 0.073 |
| No | 21 | 103 |  | 18 | 107 |  |
| Yes | 44 | 202 |  | 3 | 31 |  |
| Vascular convergence |  |  | 0.273 |  |  | 0.763 |
| No | 25 | 140 |  | 21 | 126 |  |
| Yes | 40 | 165 |  | 0 | 12 |  |
| Obstructive pneumonia |  |  | 0.125 |  |  | 0.336 |
| No | 62 | 272 |  | 9 | 64 |  |
| Yes | 3 | 33 |  | 12 | 74 |  |

Table S3 Inter-reader agreement for semantic feature classifications assigned by radiologists (R1 and R2), evaluated using Cohen's Kappa.

| Variables | Kappa values | | |
| --- | --- | --- | --- |
|  | R1 vs. R2 | R1 vs. Ground truth | R2 vs. Ground truth |
| Types | 0.94 | 0.97 | 0.97 |
| Nodule location | 0.92 | 0.97 | 0.95 |
| Shape | 0.88 | 0.96 | 0.92 |
| Lobulation | 0.85 | 0.91 | 0.93 |
| Spiculation | 0.90 | 0.95 | 0.94 |
| Cavitation | 0.86 | 0.95 | 0.91 |
| Vacuole | 0.92 | 0.96 | 0.96 |
| Air bronchograms | 0.84 | 0.96 | 0.87 |
| Pleural traction | 0.94 | 0.97 | 0.97 |
| Vascular convergence | 0.73 | 0.88 | 0.83 |
| Obstructive pneumonia | 0.82 | 0.89 | 0.92 |

Table S3 indicated that radiologists achieved a high level of inter-reader agreement in their assessments of most semantic features.

Table S4 Results of univariate and multivariate analysis in high-grade pattern prediction. OR represents the odds ratio.

| Variable | Univariate analysis | | Multivariate analysis | |
| --- | --- | --- | --- | --- |
|  | OR (95% CI) | p value | OR (95% CI) | p value |
| Age | 1.026 (1.003, 1.048) | 0.024 |  |  |
| Gender | 0.617 (0.407, 0.935) | 0.023 |  |  |
| Diameter | 1.935 (1.495, 2.504) | $<$0.001 | 1.736 (1.327, 2.272) | $<$0.001 |
| Types | 0.197 (0.118, 0.327) | $<$0.001 | 0.188 (0.109, 0.325) | $<$0.001 |
| Nodule location | 3.659 (1.219, 10.982) | 0.021 |  |  |
| Shape | 1.254 (0.805, 1.953) | 0.317 |  |  |
| Lobulation | 1.365 (0.879, 2.119) | 0.166 |  |  |
| Spiculation | 2.176 (1.428, 3.318) | $<$0.001 | 1.861 (1.16, 2.983) | 0.010 |
| Cavitation | 2.407 (0.938, 6.176) | 0.068 |  |  |
| Vacuole | 2.088 (1.199, 3.636) | 0.009 | 2.320 (1.246, 4.32) | 0.008 |
| Air bronchograms | 1.322 (0.825, 2.119) | 0.246 |  |  |
| Pleural traction | 2.11 (1.361, 3.274) | 0.001 |  |  |
| Vascular convergence | 0.909 (0.6, 1.378) | 0.653 |  |  |
| Obstructive pneumonia | 5.054 (1.918, 13.318) | 0.001 |  |  |

Table S5 Results of univariate and multivariate analysis in predominant subtype prediction.

| Variable | Univariate analysis | | Multivariate analysis | |
| --- | --- | --- | --- | --- |
|  | OR (95% CI) | p value | OR (95% CI) | p value |
| Age | 1.026 (0.998, 1.054) | 0.067 |  |  |
| Gender | 1.151 (0.671, 1.976) | 0.609 |  |  |
| Diameter | 1.433 (1.052, 1.951) | 0.023 |  |  |
| Types | 0.126 (0.07, 0.226) | $<$0.001 | 0.126 (0.07, 0.226) | $<$0.001 |
| Nodule location | 4.975 (0.658, 37.591) | 0.120 |  |  |
| Shape | 1.47 (0.842, 2.564) | 0.175 |  |  |
| Lobulation | 1.192 (0.679, 2.093) | 0.540 |  |  |
| Spiculation | 1.407 (0.823, 2.406) | 0.212 |  |  |
| Cavitation | 2.569 (0.59, 11.179) | 0.209 |  |  |
| Vacuole | 0.892 (0.463, 1.718) | 0.733 |  |  |
| Air bronchograms | 2.023 (1.011, 4.048) | 0.047 |  |  |
| Pleural traction | 0.936 (0.529, 1.658) | 0.821 |  |  |
| Vascular convergence | 0.737 (0.426, 1.274) | 0.274 |  |  |
| Obstructive pneumonia | 2.507 (0.745, 8.439) | 0.138 |  |  |

Table S6 Results of univariate and multivariate ordinal analysis for IASLC grading.

| Variable | Univariate analysis | | Multivariate analysis | |
| --- | --- | --- | --- | --- |
|  | OR (95% CI) | p value | OR (95% CI) | p value |
| Age | 1.323(1.062-1.632) | 0.011 |  |  |
| Gender | 1.502(1.003-2.250) | 0.048 |  |  |
| Diameter | 1.835(1.436-2.344) | $<$0.001 |  |  |
| Types | 8.989(5.458-14.806) | $<$0.001 | 8.989(5.458-14.806) | $<$0.001 |
| Nodule location | 0.261(0.086-0.793) | 0.018 |  |  |
| Shape | 0.743(0.484-1.140) | 0.174 |  |  |
| Lobulation | 0.729(0.477-1.115) | 0.145 |  |  |
| Spiculation | 0.450(0.299-0.678) | $<$0.001 |  |  |
| Cavitation | 0.387(0.149-1.002) | 0.051 |  |  |
| Vacuole | 0.520(0.303-0.895) | 0.018 |  |  |
| Air bronchograms | 0.651(0.408-1.039) | 0.072 |  |  |
| Pleural traction | 0.497(0.326-0.758) | 0.001 |  |  |
| Vascular convergence | 1.169(0.780-1.752) | 0.450 |  |  |
| Obstructive pneumonia | 0.191(0.072-0.508) | 0.001 |  |  |

In the univariate analyses, we use of p-value < 0.1 for feature selection may have potential pitfalls. A more lenient threshold can sometimes help avoid missing features that could be predictive of IASLC grading. On the other hand, using a smaller threshold might result in overly stringent feature selection, leading to the exclusion of potentially relevant features. Therefore, we chose a p-value threshold of 0.1 for this analysis based on the tradeoff between maximizing feature selection and minimizing the risk of missing valuable information.

Besides, understanding predictive factors might be helpful to improve model interpretability for clinical use. In the prediction of high-grade patterns, diameter, types, spiculation, and vacuole were identified as risk variables. The odds ratio for diameter was positively correlated, indicating that pulmonary lesions with larger diameters were often associated with higher risk. The results for types showed that solid pulmonary adenocarcinoma was associated with high-grade patterns. This is consistent with clinical observations, where the solid and micropapillary subtypes of pulmonary adenocarcinoma are more commonly found. Additionally, spiculation and vacuole both indicated a higher likelihood of high-grade pulmonary adenocarcinoma. In the prediction of predominant subtypes, types was the only risk variable, suggesting that acinar/papillary predominant types were more likely to present as solid lesions. In the ordinal analysis for IASLC grading, the results showed that non-solid nodules were correlated with IASLC grade I, which aligns with the findings from the prediction of high-grade patterns and predominant subtypes. For radiomics features, firstorder_Skewness and firstorder_RobustMeanAbsoluteDeviation were overlapping risk variables across radiomics models. Firstorder_Skewness measured the asymmetry of the lesion distribution. A lower skewness value suggested a more regular lesion characteristic, which is likely to correspond to lower-grade lesions. In contrast, firstorder_RobustMeanAbsoluteDeviation represented the heterogeneity of data. Higher values of this feature indicating greater heterogeneity were associated with higher-grade lesions.

In the two-step model using radiomics features, the Rad-score equation for predicting high-grade patterns was as follows: Rad_score = 0.0853 * exponential_firstorder_RobustMeanAbsoluteDeviation - 0.2463 * lbp-2D_firstorder_Entropy - 0.0480 * logarithm_ngtdm_Strength + 0.1420 * wavelet-LLL_gldm_LargeDependenceHighGrayLevelEmphasis

In the two-step model using radiomics features, the Rad-score equation for predicting predominant types was as follows: Rad_score = 0.0323 * exponential_firstorder_RobustMeanAbsoluteDeviation - 0.0186 * logarithm_firstorder_Skewness - 0.0360 * logarithm_glcm_DifferenceAverage - 0.1779 * logarithm_glcm_Idn - 0.0434 * square_gldm_LargeDependenceLowGrayLevelEmphasis + 0.0731 * wavelet-LLL_gldm_LargeDependenceHighGrayLevelEmphasis

In the one-step model using radiomics features, the Rad-score equation for directly assessing IASLC grades was as follows: Rad_score = 0.2239 * exponential_firstorder_RobustMeanAbsoluteDeviation - 0.1549 * logarithm_firstorder_Skewness + 0.1495 * wavelet-LLL_glrlm_HighGrayLevelRunEmphasis

Table S7 Effect of CT acquisition variability on the model performance

| Vendor | Slice thickness | Spacing | Total # | IASLC grading | Training AUC | Training F1 scores | Validation AUC | Validation F1 scores |
| --- | --- | --- | --- | --- | --- | --- | --- | --- |
| GE | 1.25 | 0.984 | 228 | 1 | 0.98 | 0.75 | 0.95 | 0.64 |
|  |  |  |  | 2 | 0.90 | 0.60 | 0.86 | 0.44 |
|  |  |  |  | 3 | 0.96 | 0.91 | 0.96 | 0.90 |
| Siemens | 1.5 | 0.95 | 301 | 1 | 0.94 | 0.56 | 0.96 | 0.42 |
|  |  |  |  | 2 | 0.86 | 0.56 | 0.84 | 0.46 |
|  |  |  |  | 3 | 0.95 | 0.87 | 0.97 | 0.90 |

The first-order statistics of firstorder_Skewness was mentioned in our study and another study[17], which may indicate its robustness in IASLC grading. Wavelet-related features were noted in both, but with different specifications, such as wavelet_HHH_glszm_LargeAreaEmphasis vs. wavelet-LLL_glrlm_HighGrayLevelRunEmphasis. This difference may be explained by the effects of varying vendors and imaging reconstruction parameters.


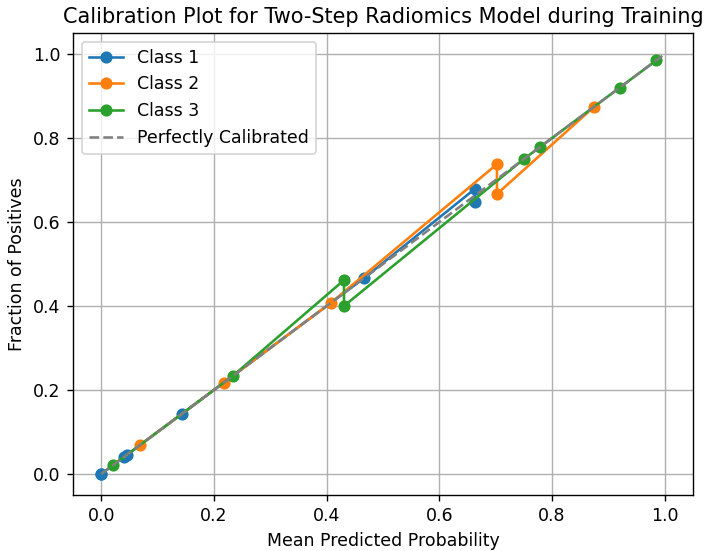

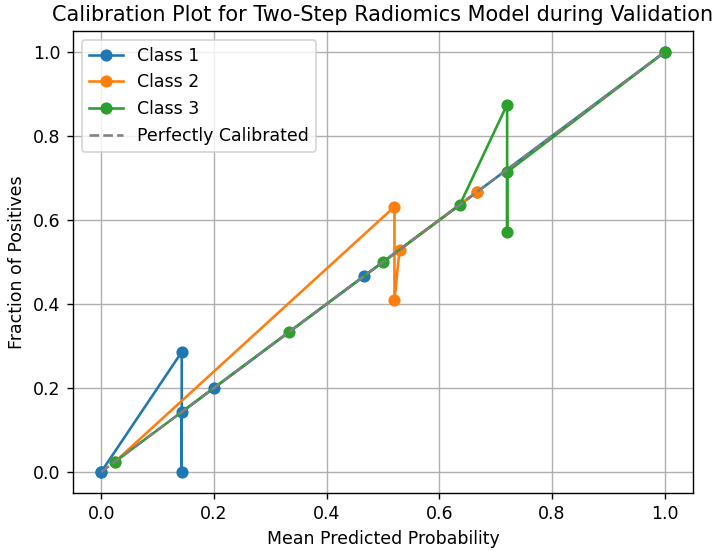


Figure S1 Calibration plots for the two-step radiomics model on the training and validation sets.

The figure shows that our two-step radiomics model predicted probabilities which were in agreement with the actual observed frequencies. This may suggest that the model was well-calibrated, providing reliable probability estimates that reflected the true likelihood of each class.
